# Circulating tumor DNA applications in monitoring the treatment of metastatic colorectal cancer patients 

**Published:** 2019

**Authors:** Nesa Kazemifard, Amir Sadeghi, Behnaz Varaminian, Hamid Rezvani, Ahmad Ayadi, Ramin Talaie, Arfa Moshiri

**Affiliations:** 1 *Gastroenterology and Liver Diseases Research Center, Research Institute for Gastroenterology and Liver Diseases, Shahid Beheshti University of Medical Sciences, Tehran, Iran *; 2 *Department of Hematology and Medical Oncology, Shahid Beheshti University of Medical Sciences, Tehran, Iran *; 3 *Gastroenterology Internal Ward, Modarres Hospital, Shahid Beheshti University of Medical Sciences, Tehran, Iran*; 4 *Basic and Molecular Epidemiology of Gastrointestinal Disorders Research Center, Research Institute for Gastroenterology and Liver Diseases, Shahid Beheshti University of Medical Sciences, Tehran, Iran*; 5 *Laboratory of Experimental Therapies in Oncology, IRCCS Istituto Giannina Gaslini, Genova, Italy*

**Keywords:** Circulating tumor DNA, Colorectal cancer, Treatment monitoring.

## Abstract

Colorectal cancer is the third most common cancer worldwide. New cancer treatment strategies such as monoclonal antibodies against growth factor and angiogenesis receptors have improved the overall survival (OS) and progression-free survival (PFS) in metastatic colorectal cancer (mCRC) patients. However, acquired resistance could happen after these therapies. Circulating tumor DNA (ctDNA) is the DNA fraction derived from tumor cells which could be applied as a non-invasive method for detecting tumor mutations before, during, and after therapies. Here, we reviewed most of the studies examining ctDNA as treatment monitoring in mCRC patients who receive different target therapies. Also, we compared ctDNA with other existing cancer-treatment monitoring methods.

## Introduction

 Colorectal cancer (CRC) has been known as the third most prevalent cancer throughout the world. Although new therapies such as anti-epithelial growth factor receptor (EGFR) and anti-vascular endothelial growth factor (VEGF) have resulted in improved metastatic CRC (mCRC) survival, primary and acquired resistance against such therapeutic drugs could still occur (1). These therapies are expensive, and patients may suffer from their toxicities and complications. Therefore, their follow-up before and during the therapy is an important necessity (2). Circulating tumor DNA (ctDNA) is cfDNA fraction arisen from tumor cells, which is diagnosed in the bloodstream of patients with cancer which is due to apoptosis, necrosis, and release of active tumor cells (3). ctDNA enables a noninvasive and repetitive analysis to gain insights into the tumor’s mutational profile and has been applied to manage personalized cancer conditions (4). Use of ctDNA for mutation analyses in various blood samples during therapy has a high potential for improving disease monitoring (4,5). Various methods have been introduced to accommodate the increasing demand for an applicable method for ctDNA analysis in clinical settings. Improving follow-up techniques for metastatic CRC patients under therapy is vital to ensure the response to therapy. The image-based Response Evaluation Criteria in Solid Tumors (RECIST) has been employed to determine the proper tumor load for treatment and also to measure the response during treatment. However, it suffers several restrictions, such as weak inter/intra-observer reproducibility and no various categorization. Further, radiographic evaluation is costly and time-consuming, which can result in accumulated ionizing radiation, while it is merely conducted once in six to eight weeks (6). Furthermore, in addition to radiographic evaluation, carcinoembryonic antigen (CEA) has been usually tested as a tumor marker for CRC. CEA has been characterized by restricted sensitivity as well as specificity since CEA concentrations increase among various other malignancies and benign tumors and are not a decisive factor in assessing tumor response/progression (7). 

Nowadays, tissue-based mutational analysis is a gold standard method for detecting resistance to targeted therapy, but it has some limitations too. It is an invasive method and not repeatable during treatment, and cannot detect all mutations because of tumor heterogeneity (8). However, liquid biopsy can address tumor heterogeneity as well as treatment-induced dynamic changes in molecular profiles online (9). In this review, we have summarized some recent investigations about ctDNA application in detecting resistance to different target therapies in mCRC patients and compared its specificity as well as sensitivity to other existing methods.

**Anti-EGFR therapy**

Epithelial growth factor receptor (EGFR) activates several signaling routes, such as RAS/MAPK and PI3K/AKT that promote cell proliferation and growth. Expression of EGF receptor increases in 82% of mCRCs (10). Cetuximab is an IgG1 chimer monoclonal antibody which can prevent the dimerization of EGFR via covering antigen epitope in domain III of EGFR and can inhibit ligand binding and EGF signaling. Further, Cetuximab can stimulate receptor internalization as well as degradation, which possibly induce an antitumor antibody‐dependent cell‐mediated cytotoxicity response (11). Panitumumab is another monoclonal antibody (mAb) which is fully humanized, and targets the extracellular domain of EGFR too. It prevents the activation of the EGFR downstream signaling cascade (12). Cetuximab and panitumumab as anti-EGFR agents have proved more effective than chemotherapy alone to treat mCRC cases with wild type (WT) KRAS gene (13). *KRAS* mutation is a valid predictive biomarker for mCRC cases resistant to anti–EGFR therapy. Specifically, patients with exon 2 KRAS mutation do not respond to anti-EGFR therapy, and they also possibly show unfavorable responses when it is associated with an oxaliplatin-based chemotherapy regimen (14). Thus, through genetic analysis of ctDNA, additional RAS mutations can be identified thereby improving patients’ selection for anti-EGFR treatments. Developing an acquired resistance at early courses of treatment is mainly caused by RAS, BRAF, and EGFR mutations (15).

In 2015, Seung Tae Kim et al. undertook a blinded study for sequencing ctDNA fragments. They used 54-gene NGS panel for 61 cases with metastatic cancer as well as 14 stage II CRC cases. Concordance between tumor DNA and ctDNA was reported 85.9%, and they detected the emergence of new KRAS resistance mutation in cetuximab-treated mCRC patients in plasma samples 1.5 months earlier than imaging (16). In another study on mCRC patients, as a first-line treatment, they received combined chemotherapy and targeted therapy. Among most cases with progressive disease, the ratio of ctDNA decreased when treatment started, while an increase was observed as treatment continued. Concordance between tissue and plasma was reported over 80% (6). In another case report, a 37-year-old woman received cetuximab with wildtype for RAS and BRAF mutations, who was diagnosed with a rectal adenocarcinoma with metastases. After a while, KRAS exon 3 mutations were recognizable using ctDNA in the plasma and increased at the time of resistance, and later a follow-up imaging indicated the disease progression. CEA and CA19-9 did not show an elevation during the assessment time (9). In another study patients with RAS and BRAF wildtype profiles received cetuximab or panitumumab treatment for the disease progression (PD). For half of patients, liquid biopsies were available at time of resistance and analyzed by droplet digital PCR. In 40% cases, ctDNA analysis results were fully concordant with the gold standard. In a patient, ctDNA could reveal some unrecognized resistance strategies in tissue biopsy and showed mutated KRAS and amplified HER2 for mechanism of resistance. On the other hand, in some patients the resistance mechanisms observed on tissue analysis could not be detected on liquid biopsies; no different and additional molecular alterations in ctDNA could be detected either (2). Jian-Ming et al. used targeted amplicon ultra-deep sequencing approach to analyzing ctDNA in mCRC cases and characterized it by acquired resistance to cetuximab; so they selected 20 mutations that are frequent in resistance mechanism, with most of these mutations being detectable in patients with acquired resistance. However, none of these mutations were identified in about 50% of patients (17). Takeshi Yamada et al. administered EGFR blockade to KRAS tumor wild type mCRC patients. They detected a new KRAS mutation as mechanism of resistance in five patients with normal CEA and CA19-9 through chemotherapy using EGFR blockade before the progression of the disease was recognizable by imaging (4). In another experiment, Plasma ctDNA and DNA from tissue samples from CRC patients were amplified using PCR. Anti-EGFR antibodies treated patients showed ctDNA, in which RAS mutations could be found prior to EGFR extracellular domain (EGFR ECD) variants. All EGFR ECD mutations were found in blood specimens after being exposed to EGFR blockade as a mechanism of resistance (18). Mariangela Russo et al. also studied EGFR blockade in colorectal cancer. A K57T MEK1 mutation was observed by analyzing a tissue biopsy from patients' responses to cetuximab, which is a new strategy for acquired resistance. In liquid samples during therapy, mutant MEK1 degrees diminished after therapy. However, an undetected KRAS mutation was observed in ctDNA and increased despite treatment. Such KRAS mutation was then recognized in another unresponsive metastasis sample (19). Morelli et al. studied plasma and tissue specimens obtained from KRAS WT mCRC cases and observed resistance against anti-EGFR monoclonal antibodies via high-sensitive emulsion RCR. ctDNA analysis indicated some detectable EGFR and KRAS mutations among 8% [0.02–0.18] and 44% [0.3–0.56] of samples, while 41% of cases showed multiple EGFR and/or KRAS mutations (20). Friederike Braig et al. scanned KRAS, NRAS, and the overlapping epitopes of EGFR antibodies (cetuximab and panitumumab) to study mutations in tumor tissue prior to as well as following therapy. In ctDNA samples, they detected a new mutation in 1 from 6 patients administrated with panitumumab, while nearly 30% of the cases demonstrated acquired RAS mutations (21). In a case report, mCRC subjects with WT KRAS were subjected to anti-EGFR therapies. In ctDNA analysis, KRAS mutation was found three months following the onset of treatment, while clinical and image progression was found two months post-treatment (22).

**Anti-VEGFR therapy**

Tumor growth, metastasis, and progression depend on angiogenesis. Angiogenesis is controlled by pro-antigenic and anti-angiogenic regulators. There is a balance between these angiogenesis regulators in healthy cells, but in cancer, this balance is impaired (23). Therapeutic agents targeting VEGFR inhibit angiogenesis and improve the patients' survival (24). Bevacizumab has been introduced as a humanized anti-VEGFR monoclonal antibody. It has been confirmed by FDA to treat different solid tumors, including CRC (25).

In a study, cfDNAs from mCRC patients were sequenced by NGS, and candidate ctDNAs were chosen by comparing them with tissue biopsies. After first-line treatment with bevacizumab, a significant reduction in the mutant allele frequency (MAF) at remission as well as enhancements in the MAF following PD were found. Masami Yamauchi et al. reported a positive relationship between MAF and tumor size as well as between reductions in MAF to less than the median score in the remission with a favorable survival rate. In two cases, new mutations were recognized in ctDNA at a low rate during post-progression survival (1).

**HER2 Blockade**

Activation of the HER2 pathway has been identified as a known resistance strategy for anti-EGFR antibody treatment, which is used as a bypass signaling pathway in first-line and salvage therapy settings (26). HER2/ErbB2/Neu is a member of tyrosine kinase receptors. Based on receptor subtype and cellular context, these signaling proteins cause several cellular processes, such as proliferation, survival, and differentiation. Activation and and amplification of HER2 receptors are linked to decreased disease-free survival in breast as well as gastro-esophagus cancers (27). Dual anti-HER2-targeted treatment (trastuzumab plus lapatinib) has shown vigorous anti-tumor activity in HER2-positive mCRC patients (28).

In a study, 33 mCRC patients were enrolled with ERBB2 amplified resistance to trastuzumab plus lapatinib treatment. Liquid biopsies were collected during treatment, and ctDNA analysis of samples was performed and compared with computed tomography (CT) scan evaluations. Almost all plasma samples showed ERBB2 copy number alteration (CNA). Changes in RAS/RAF were recognized pre-treatment among 86% of refractory cases; meanwhile, 14% of cases showed remission. Trunk mutant alleles were observed to be involved in primary resistance, and mutant subclones were associated with acquired resistance observed at final time points throughout the treatment. Tumor burden was investigated in ctDNA by truncal alterations correlated with clinical response in this study (29). In another trial, Fluorescence In Situ Hybridization (FISH) results were compared to ctDNA analysis for HER2 copy number by target sequencing, where the correlation was 66.7% (30).

**Kinase inhibitor therapy**

Tumors with *ALK* fusion rearrangements of *ALK *tyrosine kinase domain and varied unrelated gene partners cause constitutively MAP kinase activation, STAT3, and PI3K signaling pathways that drive tumor cell growth (31). Entrectinib is an oral selective inhibitor of the tyrosine kinases tropomyosin receptor kinases (Trk)A/B/C, c-ros oncogene 1 (ROS1), and anaplastic lymphoma kinase (ALK) with CNS properties to treat different solid tumors harboring gene fusions (32).

In a case study, patients carrying a *CAD-ALK *gene fusion received Entrectinib treatment. Plasma ctDNA was evaluated using the NGS-based IRCC-TARGET panel pre and post-therapy. It showed five *ALK *point mutations, while one of them was not found in ctDNA obtained before therapy. Entrectinib induced remarkable tumor shrinkage, but *CAD-ALK *mutation levels increased when the patient showed PD. *The *MAF of *ALK *gene increased continently in ctDNA samples until clinical progression was confirmed with radiological evaluation (5). In another case study, a mCRC patient with *LMNA–NTRK1 *rearrangement gene profile showed a significant therapy outcome using Entrectinib, but secondary resistance occurred latter. Liquid biopsies were collected during the treatment. Genetic analysis of ctDNA at time of progression displayed the presence of two single-point mutations in the catalytic domain of *NTRK1* making TRKA kinase insensitive to Entrectinib (33). 

Regorafenib has been shown as a multi-tyrosine kinase inhibitor binding to a minimum of 19 targets, such as angiogenic, stromal, and oncogenic tyrosine kinase receptors. Its adverse effects include fatigue, hand and foot skin reaction, and elevated liver function. Because of these adverse effects, those who initiate taking regorafenib have to be considered with follow-up visits to evaluate drug response (34).

In a study, tumor and baseline plasma specimens from 20 Acute CRC (aCRC) regorafenib-treated cases were analyzed by targeted sequencing and 89 tumor-specific mutations were identified, with ≥50% of them being also seen in baseline plasma. Also, Vandeputte et al. reported that the early enhanced mutated copies/mL was linked to the remarkable decrease in progression-free survival (PFS) and overall survival (OS)(35). Khurum Khan et al. designed a trial for RAS mutant mCRC patients with biopsiable metastasis sites. Liquid biopsies were collected monthly during regorafenib treatment for the progressive disease. ctDNA was evaluated regarding clonal RAS mutations using digital-droplet PCR. They reported that the reduction ion RAS mutant clones among ctDNA samples following a 8-week therapy showed favorable PFS (36). In another trial, regorafenib was administered to patients with refractory response to standard therapies in two cycles. A high level of total cfDNA and presence of KRAS mutation in ctDNA samples had an inverse correlation with PFS (37). 

*MET* oncogene is crucial for cancer development, including tumor induction by cancer stem cell synthesis, tumor progression by cell proliferation and survival, as well as drug resistance and metastasis (38).

MET amplification/copy gain occurs in some BRAF-mutated tumors such as CRC. The combined MET and BRAF prevention is associated with improvements in cases with rectal cancer including BRAFV600E and MET amplification. Daniele Oddo et al. analyzed ctDNA by exome sequencing and digital PCR at the time of progression in mCRC patients. MET hyper-amplification was detected in plasma samples which was confirmed with liver and lymph node metastatic biopsy analysis. They concluded that alterations of MET in BRAF mutant colorectal cancer cells can be a resistance mechanism in BRAF and MET inhibition therapy (39). In a case report, a wild type RAS, NRAS, and BRAF patient showed resistance to chemotherapy and anti-EGFR therapy. The patient received a combination of cabozantinib (MET inhibitor) plus panitumumab, and after 6 weeks, anti-tumor response was observed. ctDNA analysis showed MET amplification; however, tumor tissue results were negative for MET amplification (40). In another study, they found the presence of a KRAS G12C mutation as well as increased BRAF MAF in the ctDNA of refractory cases at relapse from combination therapy using BRAF and MEK inhibitors (41).

**ctDNA analysis VS carcinoembryonic antigen (CEA)**

A blood-based marker currently in use for treatment monitoring is carcinoembryonic antigen (CEA), but it has low sensitivity and specificity between 40 and 70% (42).

In a study, patients with CRC received postoperative chemotherapy whereby tumor tissues and serial liquid biopsies were analyzed by NGS. Driver gene mutations were detected in ctDNA at low MAFs from 63.6% of patients while these mutations were not detectable for others. In a patient, analysis of ctDNA indicated elevated TP53 mutation along with a novel mutation detected in liquid samples. On the other hand, CEA levels were lower than the threshold in the three tests before mortality, while, at the time of the last sampling it increased (43). Furthermore, after three years of follow up, recurrence-free survival was obtained 33% for ctDNA-positive cases and 87% for ctDNA-negative subjects; however, there was an elevation in CEA in 23% of recurred cases and in 1.5% of cases that did not have recurrence. Give patients with recurrence and elevated CEA were also ctDNA-positive, but only 45% of subjects with detectable ctDNA had increased CEA (44). Having examined plasma samples from CRC patients, Jeanne Tie et al. reported that ctDNA following chemotherapy was linked to a shorter recurrence-free survival. ctDNA was more frequently positive, but CEA levels were elevated at the time of radiologic recurrence (45). Elsewhere, 40 aCRC patients were enrolled in a study where the CEA and cell-free DNA (cfDNA) ratio was measured before and after first-line chemotherapy treatment. Both CEA and cfDNA were elevated in patients with progressive disease, but cfDNA was more sensitive for monitoring the drug response (46).

**Circulating tumor cells (CTC) VS ctDNA**

Solid tumors can release CTCs into the circulation, where these cells can be isolated from peripheral blood. Searching for CTCs can offer great insights into DNA, RNA, and protein components; however, their heterogeneity are shortage are limiting factors for their identification (47).

In Qiushi Sun et al. study, extraction of blood from CRC cases was performed and they were homogenized regarding the tumor. They extracted CTCs and ctDNA from blood samples. They reported that in CTC samples, approximately 47% of patients had one, while 4% showed two acquired mutations in the sample; meanwhile for ctDNA samples these results had about 80% concordance, but CTC analysis could detect most of these alterations earlier than ctDNA did. Compared to healthy controls, ctDNA samples had a higher DNA content. Further, 97% concordance was estimated in CTCs and ctDNA molecular signatures including homogenized tumor samples (48). In another study, blood samples were collected from 15 aCRC patients at different times during therapy. Analysis of KRAS, BRAF, and PIK3CA mutations was performed for CTCs and ctDNA. One mutation was observed among 78% of the blood specimens compared to tissue samples. Some cases showed a mutation in CTCs, which was not found in ctDNA and vice versa, but ctDNA and CTC showed similar dynamics in most of the cases (49).

**Methylation analyses in ctDNA**

Searching for epigenetic changes through body fluids has been shown as a modern alternative strategy for treatment monitoring. It is a stable and noninvasive method with a high frequency of positive detection. In the evaluated epigenetic biomarkers, DNA methylation is the most frequent marker in CRC (50).

In a study on ctDNA state, methylated *BCAT1 *as well as *IKZF1 *was evaluated through 12-month resection for CRC. Liquid biopsies were collected from CRC patients post-surgery where 16% of them had detectable ctDNA. Recurrence was diagnosed in 23 of the 138 (42% were ctDNA positive) cases with clinical follow-up after surgery. Based on multivariate analysis, post-operation ctDNA detection was linked to the augmented chance for relapse (51). Fanny Garlan et al. enrolled a prospective study on mCRC patients who received first- or second-line chemotherapy. They used picodroplet-digital PCR assays for detecting KRAS, BRAF, and TP53 genes or hypermethylation (WIF1, NPY) in ctDNA samples. They reported that cases with an elevated (>10 ng/mL) compared to low (_0.1 ng/mL) ctDNA level showed a limited overall survival and median PFS (7). Hyperplastic polyposis 1 (HPP1) in blood has shown association with a weak prognosis for those with mCRC. In a study analyzing methylated free-circulating DNA (mfcDNA) for HPP1 of 467 mCRC patients, this correlation was confirmed. Patients who had a reduction in their mfcDNA level after surgery had better PS, while patients without a change in their mfcDNA did not show favorable treatment outcomes based on the radiological staging (52). Methylated BCAT1 and IKZF1 are predictive biomarkers for colorectal cancer and nearly every cancer tissue indicating significant amounts of methylation in two genes. In another study, ctDNA samples were collected before and after surgery. ctDNA levels were correlated with stage, with the tumor load also shrank after surgery (53). 

## Conclusion

ctDNA is a noninvasive and repeatable method for treatment monitoring and cancer detection capable of detecting resistance gene mutations. CtDNA is accessible from blood or urine biopsies, but, because of its low concentration, it needs accurate extraction methods. Further, for tumors whose tissue biopsies are not accessible, ctDNA can be used to capture a tumor's genetic and epigenetic profile. Also, it is a real-time method as it does not need complicated and time-consuming procedures to analyze and report the diagnosis. When compared with other liquid markers for cancer detection and relapse, ctDNA is more specific and accurate. By finding a better method for measuring ctDNA and analyzing the whole genomic and epigenetic content, physicians will be able to detect CRC in early stages and choose the best therapy based on the patient’s mutational profile.

## Conflict of interests

The authors declare that they have no conflict of interest.

## References

[1] Yamauchi M, Urabe Y, Ono A, Miki D, Ochi H, Chayama K (2018). Serial profiling of circulating tumor DNA for optimization of anti-VEGF chemotherapy in metastatic colorectal cancer patients. Int J Cancer.

[2] Pietrantonio F, Vernieri C, Siravegna G, Mennitto A, Berenato R, Perrone F (2017). Heterogeneity of Acquired Resistance to Anti-EGFR Monoclonal Antibodies in Patients with Metastatic Colorectal Cancer. Clin Cancer Res.

[3] Osumi H, Shinozaki E, Takeda Y, Wakatsuki T, Ichimura T, Saiura A (2019). Clinical relevance of circulating tumor DNA assessed through deep sequencing in patients with metastatic colorectal cancer. Cancer Med.

[4] Yamada T, Iwai T, Takahashi G, Kan H, Koizumi M, Matsuda A (2016). Utility of KRAS mutation detection using circulating cell-free DNA from patients with colorectal cancer. Cancer Sci.

[5] Siravegna G, Sartore-Bianchi A, Mussolin B, Cassingena A, Amatu A, Novara L (2017). Tracking a CAD-ALK gene rearrangement in urine and blood of a colorectal cancer patient treated with an ALK inhibitor. Ann Oncol.

[6] Boeckx N, Op de Beeck K, Beyens M, Deschoolmeester V, Hermans C, De Clercq P (2018). Mutation and Methylation Analysis of Circulating Tumor DNA Can Be Used for Follow-up of Metastatic Colorectal Cancer Patients. Clin Colorectal Cancer.

[7] Garlan F, Laurent-Puig P, Sefrioui D, Siauve N, Didelot A, Sarafan-Vasseur N (2017). Early Evaluation of Circulating Tumor DNA as Marker of Therapeutic Efficacy in Metastatic Colorectal Cancer Patients (PLACOL Study). Clin Cancer Res.

[8] Song T, Mao F, Shi L, Xu X, Wu Z, Zhou J (2018). Urinary measurement of circulating tumor DNA for treatment monitoring and prognosis of metastatic colorectal cancer patients. Clin Chem lab Med.

[9] Trojan J, Klein-Scory S, Koch C, Schmiegel W, Baraniskin A (2017). Clinical Application of Liquid Biopsy in Targeted Therapy of Metastatic Colorectal Cancer. Case Rep Oncol Med.

[10] Miyamoto Y, Suyama K, Baba H (2017). Recent Advances in Targeting the EGFR Signaling Pathway for the Treatment of Metastatic Colorectal Cancer. Int J Mol Sci.

[11] Osumi H SE, Mashima T, Wakatsuki T, Suenaga M, Ichimura T, Ogura M (2018). Phase II trial of biweekly cetuximab and irinotecan as third‐line therapy for pretreated KRAS exon 2 wild‐type colorectal cancer. Cancer Sci.

[12] Battaglin F, Ahcene Djaballah S, Lenz HJ (2019). The impact of panitumumab treatment on survival and quality of life in patients with RAS wild-type metastatic colorectal cancer. Cancer Manag Res.

[13] Passardi A, Gelsomino F, Palladino MA, Casadei Gardini A, Turci D, Chiuri VE (2017). Impact of second-line cetuximabcontaining therapy in patients with KRAS wild-type metastatic colorectal cancer: results from the ITACa randomized clinical trial. Sci Rep.

[14] Douillard JY, Siena S, Tabernero J, Burkes R, Barugel M, Humblet Y (2013). Panitumumab–FOLFOX4 treatment and ras mutations in colorectal cancer. N Engl J Med.

[15] Van Helden EJ, Angus L, Menke-van der Houven van Oordt CW, Heideman DAM, Boon E, van Es SC (2019). RAS and BRAF mutations in cell-free DNA are predictive for outcome of cetuximab monotherapy in patients with tissue-tested RAS wild-type advanced colorectal cancer. Mol Oncol.

[16] Kim ST, Lee WS, Lanman RB, Mortimer S, Zill OA, Kim KM (2015). Prospective blinded study of somatic mutation detection in cell-free DNA utilizing a targeted 54-gene next generation sequencing panel in metastatic solid tumor patients. Oncotarget.

[17] Xu JM, Wang Y, Wang YL, Wang Y, Liu T, Ni M (2017). PIK3CA Mutations Contribute to Acquired Cetuximab Resistance in Patients with Metastatic Colorectal Cancer. Clin Cancer Res.

[18] Van Emburgh BO, Arena S, Siravegna G, Lazzari L, Crisafulli G, Corti G (2016). Acquired RAS or EGFR mutations and duration of response to EGFR blockade in colorectal cancer. Nat Commun.

[19] Russo M, Siravegna G, Blaszkowsky LS, Corti G, Crisafulli G, Ahronian LG (2016). Tumor Heterogeneity and Lesion-Specific Response to Targeted Therapy in Colorectal Cancer. Cancer Discov.

[20] Morelli MP, Overman MJ, Dasari A, Kazmi SM, Mazard T, Vilar E (2015). Characterizing the patterns of clonal selection in circulating tumor DNA from patients with colorectal cancer refractory to anti-EGFR treatment. Ann Oncol..

[21] Braig F, Marz M, Schieferdecker A, Schulte A, Voigt M, Stein A (2015). Epidermal growth factor receptor mutation mediates cross-resistance to panitumumab and cetuximab in gastrointestinal cancer. Oncotarget.

[22] Knebel FH, Bettoni F, da Fonseca LG, Camargo AA, Sabbaga J, Jardim DL (2019). Circulating Tumor DNA Detection in the Management of Anti-EGFR Therapy for Advanced Colorectal Cancer. Front Oncol.

[23] Qin S, Li A, Yi M, Yu S, Zhang M, Wu K (2019). Recent advances on anti-angiogenesis receptor tyrosine kinase inhibitors in cancer therapy. J Hematol Oncol.

[24] Battaglin F, Puccini A, Intini R, Schirripa M, Ferro A, Bergamo F (2018). The role of tumor angiogenesis as a therapeutic target in colorectal cancer. Expert Rev Anticancer Ther.

[25] Roviello G, Bachelot T, Hudis CA, Curigliano G, Reynolds AR, Petrioli R (2017). The role of bevacizumab in solid tumours: A literature based meta-analysis of randomised trials. Eur J Cancer.

[26] Greally M, Kelly CM, Cercek A (2018). HER2: An emerging target in colorectal cancer. Curr Probl Cancer.

[27] Connell CM, Doherty GJ (2017). Activating HER2 mutations as emerging targets in multiple solid cancers. ESMO Open.

[28] Kanat O, Ertas H, Caner B (2018). Dual HER2 inhibition strategies in the management of treatment-refractory metastatic colorectal cancer: History and status. World J Clin Cases.

[29] Siravegna G, Lazzari L, Crisafulli G, Sartore-Bianchi A, Mussolin B, Cassingena A (2018). Radiologic and Genomic Evolution of Individual Metastases during HER2 Blockade in Colorectal Cancer. Cancer Cell.

[30] Wei Q, Zhang Y, Gao J, Li J, Li J, Li Y (2019). Clinicopathologic characteristics of HER2-positive metastatic colorectal cancer and detection of HER2 in plasma circulating tumor DNA. Clin Colorectal Cancer.

[31] Yakirevich E, Resnick MB, Mangray S, Wheeler M, Jackson CL, Lombardo KA (2016). Oncogenic ALK fusion in rare and aggressive subtype of colorectal adenocarcinoma as a potential therapeutic target. Clin Cancer Res.

[32] Al-Salama ZT, Keam SJ (2019). Entrectinib: first global approval. Drugs.

[33] Russo M, Misale S, Wei G, Siravegna G, Crisafulli G, Lazzari L (2016). Acquired resistance to the TRK inhibitor entrectinib in colorectal cancer. Cancer Discov.

[34] Byrne M, Saif MW (2019). Selecting treatment options in refractory metastatic colorectal cancer. Onco Targets Ther.

[35] Vandeputte C, Kehagias P, El Housni H, Ameye L, Laes JF, Desmedt C (2018). Circulating tumor DNA in early response assessment and monitoring of advanced colorectal cancer treated with a multi-kinase inhibitor. Oncotarget.

[36] Khan K, Rata M, Cunningham D, Koh DM, Tunariu N, Hahne JC (2018). Functional imaging and circulating biomarkers of response to regorafenib in treatment-refractory metastatic colorectal cancer patients in a prospective phase II study. Gut.

[37] Wong AL, Lim JS, Sinha A, Gopinathan A, Lim R, Tan CS (2015). Tumour pharmacodynamics and circulating cell free DNA in patients with refractory colorectal carcinoma treated with regorafenib. J Transl Med.

[38] Zhang J, Babic A (2016). Regulation of the MET oncogene: molecular mechanisms. Carcinogenesis.

[39] Oddo D, Siravegna G, Gloghini A, Vernieri C, Mussolin B, Morano F (2017). Emergence of MET hyper-amplification at progression to MET and BRAF inhibition in colorectal cancer. Br J Cancer.

[40] Jia J, Morse MA, Nagy RJ, Lanman RB, Strickler JH (2018). cell-free DNA profiling to discover mechanisms of exceptional response to cabozantinib plus panitumumab in a patient with treatment refractory metastatic colorectal cancer. Front Oncol.

[41] Oddo D, Sennott EM, Barault L, Valtorta E, Arena S, Cassingena A (2016). Molecular landscape of acquired resistance to targeted therapy combinations in BRAF-mutant colorectal cancer. Cancer Res.

[42] Nikolaou S, Qiu S, Fiorentino F, Rasheed S, Tekkis P, Kontovounisios C (2018). Systematic review of blood diagnostic markers in colorectal cancer. Tech Coloproctol.

[43] Sun X, Huang T, Cheng F, Huang K, Liu M, He W (2018). Monitoring colorectal cancer following surgery using plasma circulating tumor DNA. Oncol Lett.

[44] Tie J, Cohen JD, Wang Y, Li L, Christie M, Simons K (2019). Serial circulating tumour DNA analysis during multimodality treatment of locally advanced rectal cancer: a prospective biomarker study. Gut.

[45] Tie J, Wang Y, Tomasetti C, Li L, Springer S, Kinde I (2016). Circulating tumor DNA analysis detects minimal residual disease and predicts recurrence in patients with stage II colon cancer. Sci Transl Med.

[46] Yu D, An G, Xu L (2016). Investigation of Efficacy Evaluation Comparison of cfDNA and CEA in Colorectal Cancer. Clin Lab.

[47] Yamada T, Matsuda A, Koizumi M, Shinji S, Takahashi G, Iwai T (2019). Liquid Biopsy for the Management of Patients with Colorectal Cancer. Digestion.

[48] Sun Q, Liu Y, Liu B, Liu Y (2018). Use of Liquid Biopsy in Monitoring Colorectal Cancer Progression Shows Strong Clinical Correlation. Am J Med Sci.

[49] Kidess-Sigal E, Liu HE, Triboulet MM, Che J, Ramani VC, Visser BC (2016). Enumeration and targeted analysis of KRAS, BRAF and PIK3CA mutations in CTCs captured by a label-free platform: Comparison to ctDNA and tissue in metastatic colorectal cancer. Oncotarget..

[50] Ma Z, Williams M, Cheng YY, Leung WK (2019). Roles of Methylated DNA Biomarkers in Patients with Colorectal Cancer. Dis Markers.

[51] Murray DH, Symonds EL, Young GP, Byrne S, Rabbitt P, Roy A (2018). Relationship between post-surgery detection of methylated circulating tumor DNA with risk of residual disease and recurrence-free survival. J Cancer Res Clin Oncol.

[52] Herbst A, Vdovin N, Gacesa S, Ofner A, Philipp A, Nagel D (2017). Methylated free-circulating HPP1 DNA is an early response marker in patients with metastatic colorectal cancer. Int J Cancer.

[53] Symonds EL, Pedersen SK, Murray DH, Jedi M, Byrne SE, Rabbitt P (2018). Circulating tumour DNA for monitoring colorectal cancer-a prospective cohort study to assess relationship to tissue methylation, cancer characteristics and surgical resection. Clin Epigenetics.

